# MEF2 transcription factors differentially contribute to retinal ganglion cell loss after optic nerve injury

**DOI:** 10.1371/journal.pone.0242884

**Published:** 2020-12-14

**Authors:** Xin Xia, Caroline Y. Yu, Minjuan Bian, Catalina B. Sun, Bogdan Tanasa, Kun-Che Chang, Dawn M. Bruffett, Hrishikesh Thakur, Sahil H. Shah, Cara Knasel, Evan G. Cameron, Michael S. Kapiloff, Jeffrey L. Goldberg

**Affiliations:** 1 Mary M. and Sash A. Spencer Center for Vision Research, Department of Ophthalmology, Byers Eye Institute, Stanford University School of Medicine, Palo Alto, CA, United States of America; 2 Department of Medicine and Stanford Cardiovascular Institute, Stanford University School of Medicine, Palo Alto, CA, United States of America; National Eye Centre, UNITED STATES

## Abstract

Loss of retinal ganglion cells (RGCs) in optic neuropathies results in permanent partial or complete blindness. Myocyte enhancer factor 2 (MEF2) transcription factors have been shown to play a pivotal role in neuronal systems, and in particular MEF2A knockout was shown to enhance RGC survival after optic nerve crush injury. Here we expanded these prior data to study bi-allelic, tri-allelic and heterozygous allele deletion. We observed that deletion of all MEF2A, MEF2C, and MEF2D alleles had no effect on RGC survival during development. Our extended experiments suggest that the majority of the neuroprotective effect was conferred by complete deletion of MEF2A but that MEF2D knockout, although not sufficient to increase RGC survival on its own, increased the positive effect of MEF2A knockout. Conversely, MEF2A over-expression in wildtype mice worsened RGC survival after optic nerve crush. Interestingly, MEF2 transcription factors are regulated by post-translational modification, including by calcineurin-catalyzed dephosphorylation of MEF2A Ser-408 known to increase MEF2A-dependent transactivation in neurons. However, neither phospho-mimetic nor phospho-ablative mutation of MEF2A Ser-408 affected the ability of MEF2A to promote RGC death *in vivo* after optic nerve injury. Together these findings demonstrate that MEF2 gene expression opposes RGC survival following axon injury in a complex hierarchy, and further support the hypothesis that loss of or interference with MEF2A expression might be beneficial for RGC neuroprotection in diseases such as glaucoma and other optic neuropathies.

## Introduction

Retinal ganglion cell (RGC) death underlies vision loss in glaucoma, a common disease affecting ~80 million people worldwide, of whom ~10% are predicted to go blind [1]. Other ophthalmic diseases incurring loss of RGCs and blindness include ischemic optic neuropathy, optic neuritis, and trauma to the optic nerve, together prompting the search for new therapies for RGC neuroprotection. The failure of RGCs to survive and regenerate their axons after injury results in part from a lack of adequate trophic signaling by endogenous key regulatory molecules, including signaling that affects RGC gene expression [2].

The MADS (MCM-1-agamous-deficiens-serum response factor) box transcription factor MEF2 was originally defined as a muscle-specific factor, but later found to be expressed ubiquitously and to regulate many tissue phenotypes. Although MEF2 family members are widely expressed across tissues, they have isoform-specific effects in different tissues or cells that presumably depend upon differential interaction with co-regulatory factors [3]. MEF2 transcription factors have been identified as major activity-dependent regulators of neuronal development, function and survival after injury in the central neuronal system, including brain and retina [4–6]. In the developing retina, MEF2D is highly expressed in all retinal neurons, and MEF2A and MEF2D are the major isoforms expressed in RGCs [7, 8]. Although MEF2D was not required for RGC development and cell fate determination, global MEF2D knock-out resulted in selective post-natal degeneration of photoreceptor cells and vision loss [7, 8]. The unique function of MEF2D in developing photoreceptor cells was attributed to the absence of other MEF2 family members in that cell type and the co-regulation of photoreceptor-specific genes by the transcription factor CRX. The function of MEF2 proteins in RGC development remains unknown, but is presumably distinct from that in photoreceptors due to a lack of CRX in RGCs [9].

MEF2 proteins also play a critical role in the regulation of neuronal survival in response to both retrograde neurotrophin stimulation and neuronal depolarization, including in retinal ganglion cells (RGCs) [10–13]. MEF2 is typically considered neuroprotective in stress models [14, 15]. For example, inactivation of MEF2A and MEF2D by cyclin-dependent kinase 5 (Cdk5) phosphorylation was shown to promote cerebellar granule neuron apoptosis due to glutamate toxicity [16]. On the other hand, *Mef2a* gene targeting was recently shown to prevent RGC apoptosis and improve survival after optic nerve crush [17]. Here we confirm and extend these results by studying *Mef2a*, *-c* and *-d* alleles during development and after optic nerve crush, using both cre-lox targeting and overexpression of MEF2A point mutants previously linked to stress-related signaling.

## Materials and methods

### Bioinformatics analysis of RNA-seq datasets

RNA-seq datasets for before (*n* = 5), 1 day (*n* = 4) and 5 days (*n* = 4) post-optic nerve crush will be deposited and described elsewhere. Briefly, RNA-seq reads were aligned to the mouse genome mm10 using the ENCODE computational pipeline on a DNAnexus cloud computing platform that includes the STAR alignment package and RSEM counting algorithm. Differentially expressed genes were identified by using limma-voom in R/BioC, and genes with a FDR < 0.05 and an absolute value fold change (FC) greater than 1.2 were considered for subsequent analysis.

Mouse models

All animal procedures were done in accordance with The Association for Research in Vision and Ophthalmology Statement for the Use of Animals in Ophthalmic and Vision Research and approved by the Administrative Panel of Laboratory Animal Care (APLAC) Institutional Animal Care and Use Committee (IACUC) at Stanford University. *Mef2a*, *Mef2c* and *Mef2d* floxed allele mice maintained on a mixed C57BL/6 background and having different combinations of the floxed alleles were bred from a triply floxed mouse provided generously by Drs. Eric Olson and Rhonda Bassel-Duby (University of Texas, Southwestern, Dallas, TX) [18]. Cre-mediated excision in these mice result in deletion of the MEF2 MADS and DNA binding domains. Genotypes were confirmed for each animal by genomic PCR, using ear tissue, as previously described [18, 19]. Mating *Mef2a*^*f/f*^*; Mef2c*^*f/f*^*; Mef2d*^*f/f*^ mice to Tg(Chx10-EGFP/cre,-ALPP)2Clc/J (Jackson Laboratory stock #005105) mice was used to confer triple gene deletion in the entire retina during early development. 129X1/SvJ mice were used for experiments involving exogenous MEF2A cDNA expression. For all experiments, adult mice of either sex were used.

### Plasmids and AAV

Adeno-associated virus stocks were ~5 x 10^12^ viral genomes/ml as determined by quantitative alkaline gel electrophoresis. Gene deletion was conferred by injection of AAV2-Cre- green fluorescent protein (GFP) as previously described, with AAV2-GFP serving as control [20]. Flag–tagged MEF2A wild-type and mutant proteins were expressed by intravitreal injection of AAV2 generated with pAAV-CMV-Flag-Mef2A shuttle vectors constructed as follows: pEntr-Mef2aflbio containing a human MEF2A cDNA (GenBank: X68505.1) was a gift from William Pu (Addgene plasmid # 32970; RRID: Addgene_32970) [21]. The MEF2A cDNA was inserted by PCR-based cloning into an AAV shuttle plasmid containing flanking AAV2 ITRs, a CMV immediate early promoter, and SV40 polyadenylation signal and providing a Flag-tag N-terminal fusion to the MEF2A cDNA (additional details available upon request). MEF2A S408A and S408E missense mutations were introduced by site-directed mutagenesis introducing GATT**G**C**T**CCTCC**A**CGGG (new Bcg I site) and GAT**CGA**ACCTCCTCGGG (new Pvu I site), respectively.

### Intravitreal injection, optic nerve crush and anterograde labeling

Mice aged postnatal day 25–30 (P25-30) under isoflurane anesthesia were injected intravitreally through the sclera 1 mm below the limbus, being careful to avoid injury to the lens, with 1 μl AAV in PBS using a foot-pedal controlled picospritzer (PICO). Two weeks after AAV injection, mice were anesthetized with ketamine (100 mg/kg) and xylazine (20 mg/kg), and optic nerve crush was performed by cross-action forceps (Dumont, RS-5020, Roboz Surgical Instrument, Galthersburg, MD) 2 mm behind the eyeball for 3 sec, as previously described [20, 22]. Care was taken to avoid damaging the blood supply to the retina. Buprenorphine was provided for post-operative analgesia. Two weeks later, mice were intravitreally injected with 1 μL Alexa-555 cholera toxin B (1mg/mL) (Thermo Fisher Scientific, Cat #C22843) to visualize optic nerve axons and detect axonal regeneration.

### Retinal flat-mount and immunohistochemistry

Flat-mount sections were prepared two weeks after optic nerve crush as described previously [20, 22, 23]. Briefly, mice under deeply anesthesia using isoflurane induction and intraperitoneal injection of ketamine and xylazine were euthanized by transcardial perfusion. Eyes were harvested and post-fixed with 4% PFA for 1 h at room temperature. Retinas were isolated, washed with PBS in 48-well plate, permeabilized with 2% Triton^TM^-X-100 in PBS, and stained with rabbit anti-RBPMS antibody (1:4000, custom-made by ProSci, Poway, CA) overnight in blocking buffer (PBS with 10% normal goat serum, Thermo Fisher). Sections were then washed and then stained with Alexa-647 anti-guinea pig secondary antibody (1:200, Thermo Fisher Catalog #ab150187) in blocking buffer at room temperature for 1 hour, followed by mounting on slides using ProLong Gold Anti-Fade mounting medium (Thermo Fisher). 100X Tile scan images were acquired by confocal laser scanning microscopy (Airyscan 880, Zeiss).

### Quantification of RGC survival

Cell counting was performed as previously described [24]. In brief, retinas were divided into 4 quadrants. Using ImageJ (FIJI), 500 μm square areas were selected within each quadrant 1100–1200 μm away from the optic nerve head for measurement of RGC density. RBPMS-positive cells were counted manually by one experienced investigator who was masked to experimental group. Results are presented as cells/mm^2^ for each retina or survival fraction compared to sham-operated contralateral eyes.

### Statistical analysis

Gene expression data were analyzed by false discovery rate. RGC survival data were analyzed by Student’s t-test or by one-way ANOVA followed by Tukey post-hoc testing. * indicates *p* < 0.05; ** indicates *p* < 0.01.

## Results

### MEF2A, MEF2C and MEF2D are not require for RGC development

**Fig 1:**
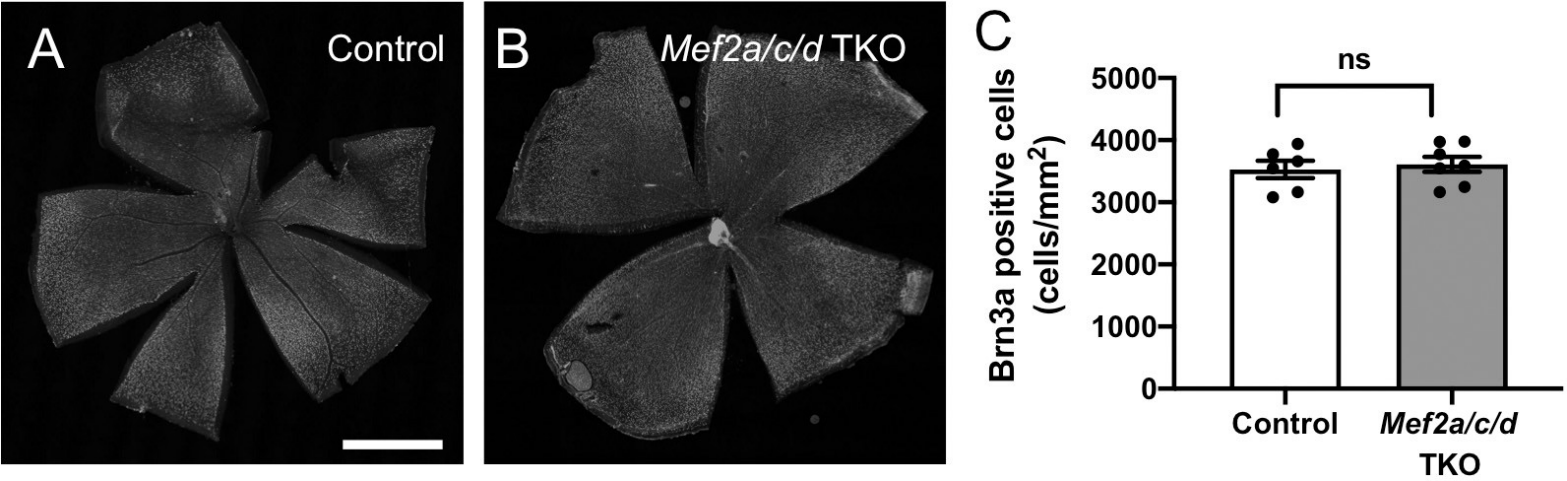
MEF2 is not required for RGC development. (A, B) Flat-mounted retinas from control and *Mef2a/c/d* mice (TKO) triply targeted using a Chx10-cre driver, immunostained against Brn3a (scale bar, 1 mm). (C) Quantification of Brn3a-positive cells showed no detectable difference in RGC numbers through development.

To determine if MEF2 family members are relevant to RGC differentiation or survival in retinal development, mice containing floxed alleles for *Mef2a*, *Mef2c*, and *Mef2d* were mated to a Chx10-cre recombinase driver line [25] to create a triple knock-out mouse (*Mef2a/c/d* TKO) lacking MEF2A/C/D in retinal progenitors and their progeny from early embryonic development. Retinas isolated from 38-day old mice (P38) were immunostained with the RGC-specific marker Brn3a (Fig 1A and 1B). There was no significant difference in RGC number between wild-type and *Mef2a/c/d* TKO mice (Fig 1C), demonstrating that MEF2 transcription factors are not required for RGC differentiation or survival into adulthood.

### MEF2 gene expression in response to optic nerve crush injury

**Fig 2:**
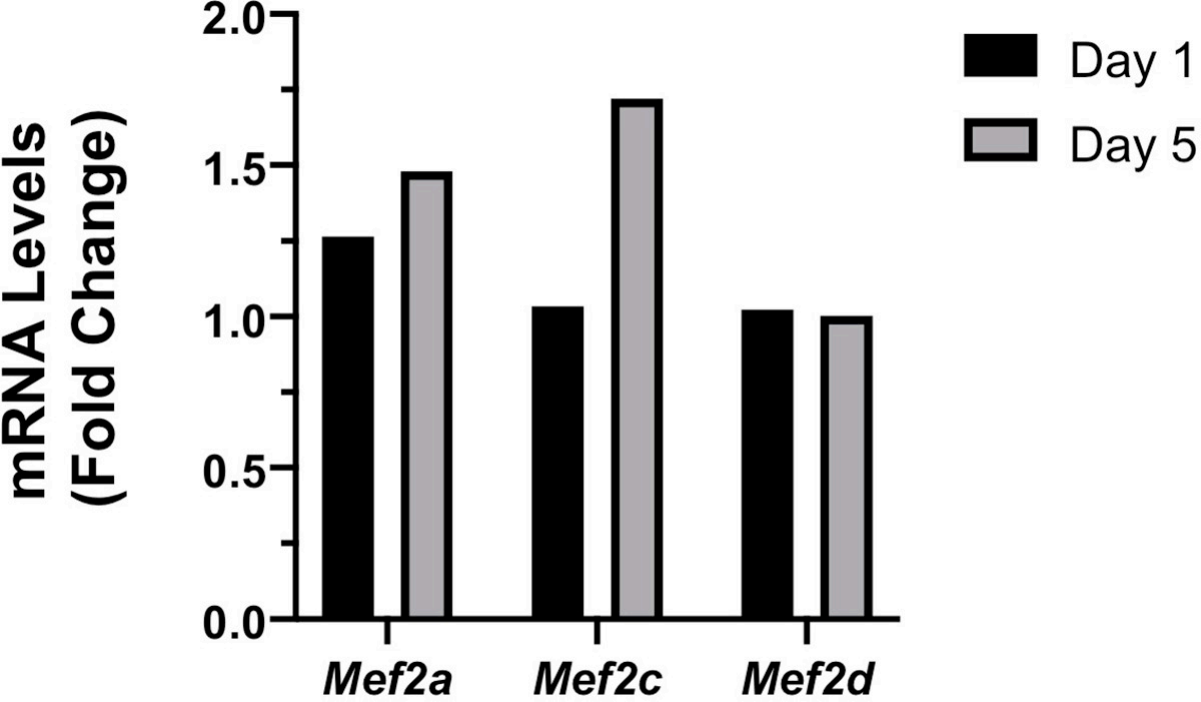
*Mef2* gene expression after optic nerve crush. Fold-change gene expression was determined from RNA-seq datasets for control (non-crushed, *n* = 5), 1 day post-crush (*n* = 4) and 5 days post-crush (*n* = 4) purified RGCs. At 5 days post-crush, *Mef2a* and *Mef2c* mRNA levels were increased 1.5 (FDR = 0.02) and 1.7-fold (FDR = 0.06), respectively, while expression of *Mef2d* was not changed (FDR = 0.99). *Mef2b* expression was not detected in RGCs.

mRNA for *Mef2a* and *Mef2d*, but not *Mef2b* and *Mef2c* are readily detected by *in situ hybridization* in RGCs in the mature retina [8], albeit weak detection of *Mef2c* protein has been reported in mouse, but not human RGCs [26]. Using RNA-seq datasets derived from RGCs purified at 1 and 5 days after optic nerve crush (The accession number for GEO dataset is GSE142881), we found that mRNA levels for *Mef2a* and *Mef2c* were increased 1.5- and 1.7-fold, respectively (FDR = 0.02 and = 0.06, respectively) 5 days after optic nerve crush injury (Fig 2). mRNA levels for *Mef2b* were not detected in RGCs either before or after injury, and the relatively higher levels of *Mef2d* mRNA were not affected by optic nerve crush injury.

### *Mef2a* gene targeting confers RGC neuroprotection

**Fig 3:**
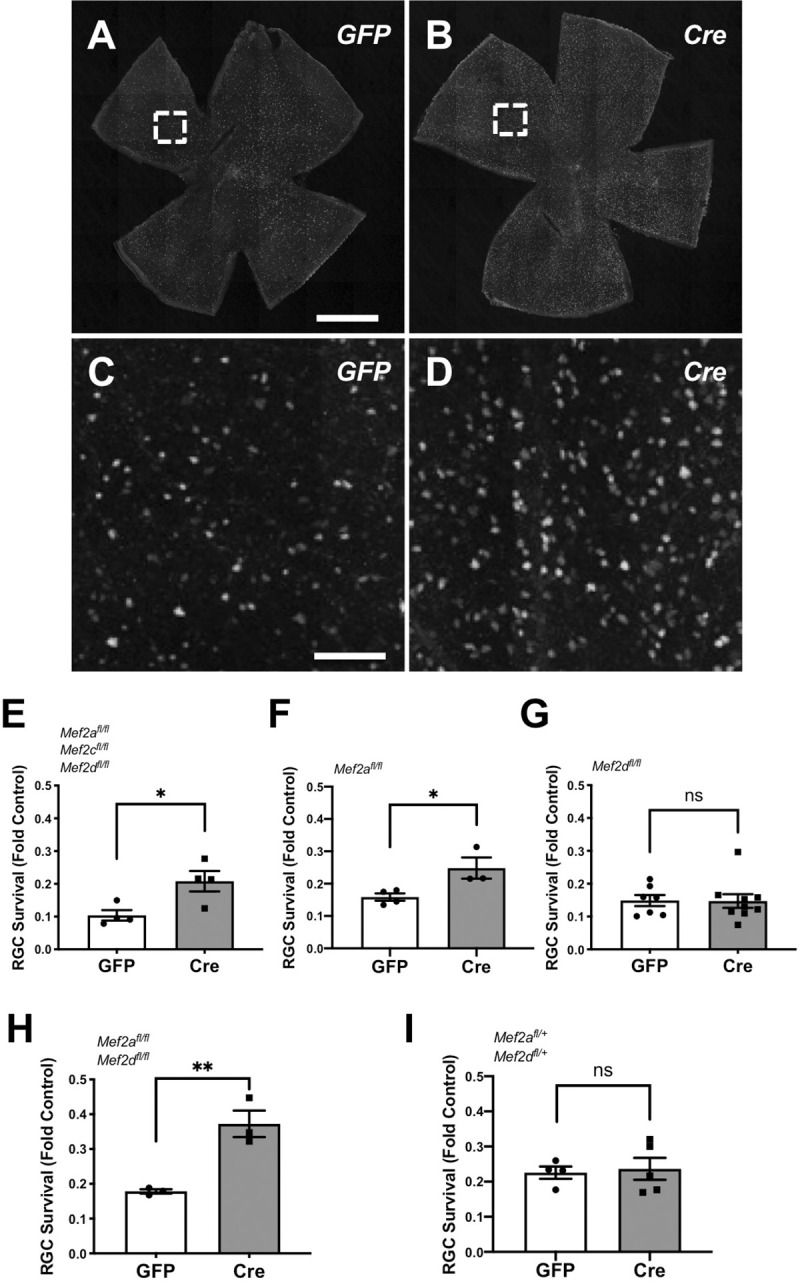
*Mef2a* gene targeting promotes RGC survival after optic nerve crush. (A-D) Representative images of RBPMS-positive RGCs from *Mef2a*^*f/f*^;*Mef2d*^*f/f*^ mice injected with either AAV2-cre-GFP or AAV2-GFP control (scale bar in A, B, 1 mm; in C, D, 100 μm). (E-I) Mice of the indicated genotypes injected with either AAV2-cre-GFP or AAV-GFP control were subjected to optic nerve crush. RBPMS expressing cells were quantified two weeks later. RGC counts are normalized by the number of RGCs in the contralateral non-crushed eye. Each datapoint represents an individual mouse. Data were analyzed by Student’s *t*-test. *Mef2a* knock-out increased RGC survival 1.5-fold compared with control eyes; *Mef2a/d* double knock-out and *Mef2a/c/d* triple knock-out increased RGC survival ~2-fold compared with control eyes. *Mef2d* knock-out and hemizygous *Mef2a/d* deletion had no significant (ns) effect on RGC survival.

In order to test whether MEF2 family members confer RGC neuroprotection after optic nerve injury, we used a loss-of-function approach. Intravitreal injection of AAV2 to express cre recombinase in RGCs was used to induce conditional gene deletion in P25-P30 mice carrying one or more *Mef2a*, *Mef2c*, and *Mef2d* floxed alleles. Two weeks later, mice were subjected to optic nerve crush or sham surgery. Then, two weeks after injury, retinas were isolated and immunostained for the RGC marker RBPMS to identify surviving RGCs (Fig 3A–3D). We found that triple *Mef2a/c/d* knock-out increased the number of surviving RGCs 2 weeks after injury (Fig 3E). Targeting *Mef2a* alone also promoted RGC survival (Fig 3F), whereas targeting *Mef2d* alone showed no effect (Fig 3G). Notably, double *Mef2a/d* knock-out promoted a greater fold-change increase in RGC survival than loss of *Mef2a* alone (Fig 3H), with the caveat that control AAV2-GFP-expressing mice exhibited somewhat different baseline survival for the mice with different floxed alleles. Hemizygous knock-out of both *Mef2a* and *Mef2d* showed no effect (Fig 3I). Examination of the optic nerve for anterogradely transported fluorescent cholera toxin B (CTB) revealed no evidence of axon regeneration in any of the mouse cohorts. Thus MEF2A when present in at least 1 allele, potentially in synergy with MEF2D, contributes to RGC death after optic nerve injury, such that their loss promotes survival.

### Effects of exogenous MEF2A

**Fig 4:**
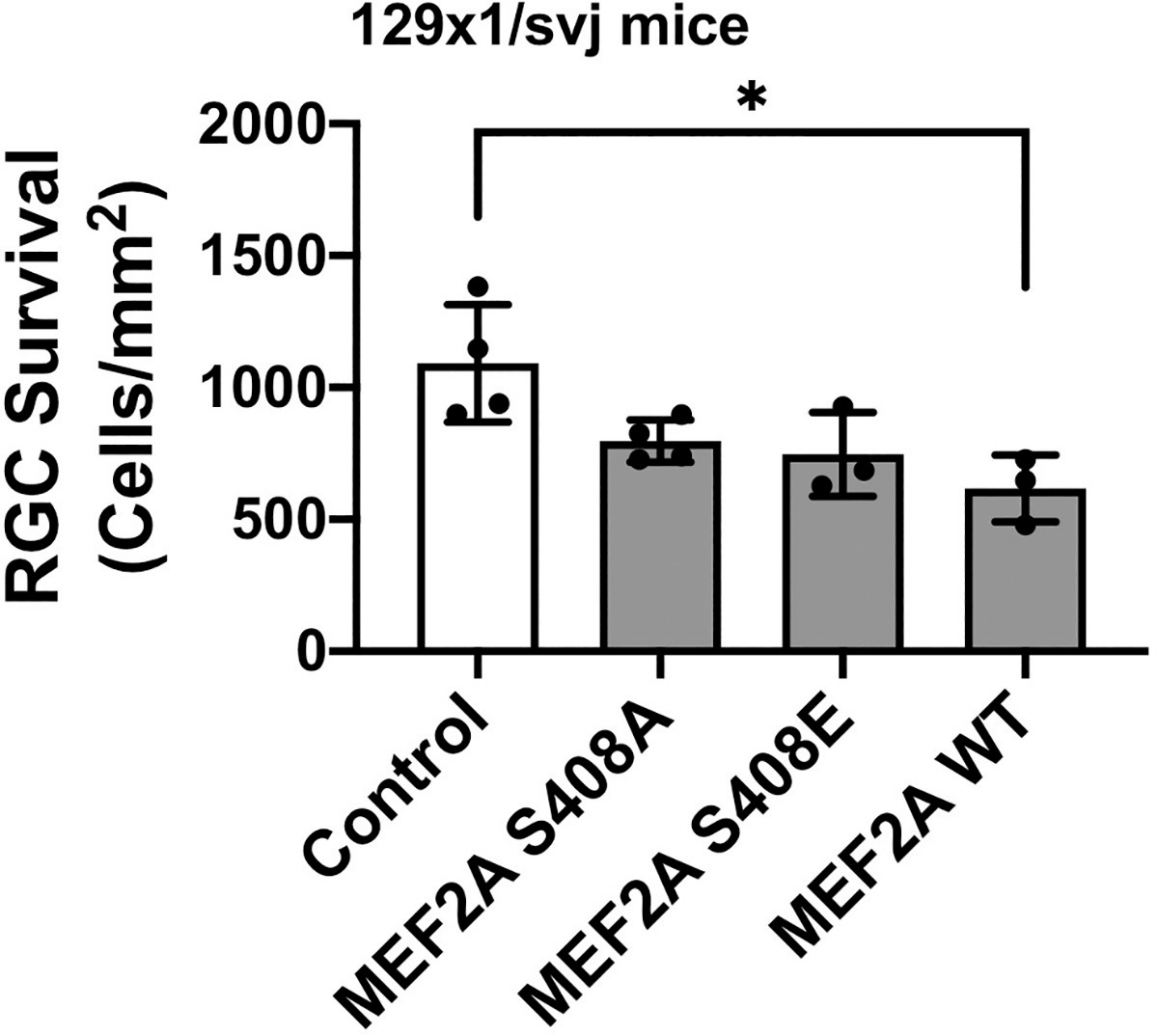
MEF2A over-expression promotes RGC loss after optic nerve crush. AAV2 expressing Flag-tagged MEF2A wildtype (WT), S408A, S408E mutants or luciferase control were injected into wild type mice two weeks before optic nerve crush. Two weeks post-crush, RPBMS-positive cells were counted following immunostaining of flat-mount retinas as in Fig 3. Data represent the absolute number of RGCs per unit area of the retina, and individual datapoints represent individual mice. Data were analyzed by one-way ANOVA and Tukey post-doc testing. Exogenous expression of wild-type Mef2a decreased RGC survival (* *p*<0.05); both S408A and S408E showed a similar trend towards decreasing RGC survival and were not statistically significantly different from Mef2a WT expression.

We next asked whether MEF2A gain-of-function would, conversely, worsen RGC survival after optic nerve crush. In addition, MEF2A Ser-408 phosphorylation is a well-established mechanism for control of MEF2A activity in cells, promoting a switch between MEF2A-dependent gene activation and repression [13, 27, 28]. Thus, flag-tagged MEF2A wildtype and mutant proteins were expressed by AAV2 intravitreal injection of wild type mice. Flag-tag immunostaining was not detected, presumably because of the RGC death promoted by MEF2A constructs. Overexpression of wildtype MEF2A decreased RGC survival by 43% (Fig 4). Surprisingly, expression of MEF2A S408A and S408E phosphomimetic and phosphoablative mutants resulted in a similar trend towards worsening of RGC survival (*p* = 0.10 and 0.07, respectively), resulting in cell death not significantly different from that induced by wild-type MEF2A expression. Again, axonal regeneration measured by anterograde CTB labeling was not detected following expression of any of the MEF2A recombinant proteins.

## Discussion

In this study, we find that MEF2A expression is deleterious for RGC survival after RGC axon injury. While MEF2D loss did not promote neuroprotection on its own, combined loss of the two factors resulted in RGC survival greater than that conferred by loss of MEF2A alone. These data confirm the recently published findings using the same *Mef2* knock-out mice that *Mef2a* and triple *Mef2a/c/d* gene targeting promote RGC survival in vitro and after optic nerve crush [17]. As recognized by both datasets, it is quite surprising that MEF2A promotes RGC loss instead of survival after optic nerve crush. Multiple prior publications demonstrate the opposite, namely, a neuroprotective function of MEF2A and MEF2D in cerebellar granule, cortical, hippocampal and dopaminergic neurons that together underscore the concept that molecular mechanisms may not be conserved among neuronal cell types [10, 14–16, 29–31]. Our findings that *Mef2a* knock-out increased RGC survival, whereas MEF2A overexpression increased RGC death, together suggest that MEF2A actively promotes cell death through either the repression of pro-survival or transactivation of pro-death gene expression.

It is surprising but not unprecedented that targeting only *Mef2d* had no effect on RGC survival. Less is known about MEF2A in the retina, but due to its hetero-dimerization with MEF2D, one might have expected that MEF2A would have overlapping functions with MEF2D in RGCs [32]. The four MEF2 family members (A-D) all bind via their MADS domain the consensus DNA motif YTAWWWWTAR [33, 34]. While not regulating identical gene sets, MEF2A and MEF2D have redundant roles in cerebellar-dependent motor learning, such that MEF2A will occupy relevant MEF2D genomic sites when MEF2D is depleted [35]. In contrast, the differing functions of MEF2A and MEF2D in RGCs is supported by our observation that loss of one allele for each of the two factors conferred no neuroprotection. On the other hand, the greater fold increased RGC survival following loss of both MEF2A and MEF2D suggests that MEF2D can partially complement MEF2A depletion.

Unexpectedly, the ability of MEF2A to oppose RGC survival was independent of canonical regulation at Ser-408. Previous work has shown that phosphorylation by Cdk5 converts MEF2 to a transcriptional repressor, whereas dephosphorylation catalyzed by the Ca^2+^/calmodulin-dependent phosphatase calcineurin (PP2B) results in transactivation of MEF2-dependent gene expression [36]. Consistent with these data, a phosphoablative mutant for MEF2A S408 had increased transcriptional activity in neurons [37]. An elegant model has been proposed for the activation of MEF2A in cerebellar neuron post-synaptic differentiation, in which calcineurin dephosphorylation of MEF2A Ser-408 promotes the desumoylation and acetylation of Lys-403, increasing MEF2A-dependent transcription [13, 28]. Interestingly, Welsbie et al reported that MEF2A Ser-408 was highly phosphorylated after optic nerve crush injury, via an unknown mechanism dependent upon expression of the pro-death dual leucine zipper kinase (DLK) [17]. Here we addressed this model directly and found that overexpression of wildtype, S408A, and S408E MEF2A all had similar deleterious effects on RGC survival, implying that this canonical switch between repression and activation is not relevant in RGCs. Interestingly, protein phosphatase Iα has been shown to bind MEF2A, strongly inhibiting MEF2A-dependent transcription via recruitment of histone deacetylase 4 [38]. The effect of protein phosphatase Iα was dominant to calcineurin activation and induced repression even in the presence of S408A mutation. Future studies will be required to determine whether alternative modes of regulation, such as by protein phosphatase Iα, are important for promoting RGC cell death, and to determine the genomic sites of action for MEF2A in opposing neuroprotection.

Together these data support a role for MEF2A-regulated gene expression that opposes RGC survival after axon injury. We previously found that expression of the perinuclear scaffold protein muscle A-kinase anchoring protein α (mAKAPα) is required for neurotrophic factor- and cyclic-adenosine mononucleotide (cAMP)-dependent RGC neuroprotection after optic nerve crush injury, but not during RGC development [20], while MEF2 regulation in myocytes is regulated by mAKAP signalosomes [27, 39]. The previous finding that MEF2A expression mediates survival signaling downstream of the stress-induced DLK/LZK pathway similarly places MEF2A at a central locus in the regulation of stress-induced survival signaling [17]. Identification of the genes regulated by MEF2A may provide new opportunities for intervention in RGC diseases.
